# Assessment of compliance and therapeutic efficacy of albendazole treatment in Chinese patients with echinococcosis

**DOI:** 10.1186/s40249-024-01268-3

**Published:** 2024-12-20

**Authors:** Min Qin, Guobing Yang, Jun Yan, Liying Wang, Yu Feng, Dong Wang, Qian Wang, Yanyan Hou, Jiangshan Zhao, Jiaxi Lei, Zhiyi Wang, Mingzhe Jiang, Chenghang Yu, Laurent Gavotte, Roger Frutos

**Affiliations:** 1 National Institute of Parasitic Diseases, Chinese Center for Disease Control and Prevention (Chinese Centre for Tropical Diseases Research); NHC Key Laboratory of Parasite and Vector Biology; WHO Collaborating Centre for Tropical Diseases, National Centre for International Research On Tropical Diseases, Shanghai, China; 2 Chaoyang District Center for Diseases Prevention and Control of Beijing, Beijing, China; 3 Gansu Provincial Center for Disease Control and Prevention, Lanzhou, China; 4 Chinese Centre for Disease Control and Prevention, Beijing, China; 5 Sichuan Provincial Center for Disease Control and Prevention, Chengdu, China; 6 Xinjiang Uygur Autonomous Region Center for Disease Control and Prevention, Urumqi, China; 7 Espace-Dev, UMR 228, Université de Montpellier, Montpellier, France; 8 Centre de Cooperation International en Recherche Agronomique Pour Le Développement, UMR 17, Intertryp, Campus International de Baillarguet, Montpellier, France; 9 Faculty of Medicine-Ramathibodi Hospital, Mahidol University, Bangkok, Thailand

**Keywords:** Albendazole, Therapy, Medication compliance, Echinococcosis, Therapeutic effect

## Abstract

**Background:**

Echinococcosis is an infectious parasitic disease that is extremely harmful to human health. Albendazole is provided free of charge to patients requiring medication under the central government finance transfer payment scheme for echinococcosis control and prevention in China. Our aim is to monitor the state of patient medication and its therapeutic impact, which will help improve medication compliance and the therapeutic effect.

**Methods:**

Random cluster sampling was used to select 10 echinococcosis-endemic counties in China, and all albendazole-treated patients in these counties were investigated. The chi-square and Kruskal–Wallis tests were used to compare two or more rates or constituent ratios, and multiple logistic regression analysis was used to identify the influencing factors. The records of patients were reviewed to obtain the initial diagnosis results as well as the most recent follow-up results and time, and efficacy was assessed.

**Results:**

We examined 899 patient files treated with albendazole in 10 endemic counties. Of the 582 evaluable files, 7.9% did not take albendazole, and 69.2% did not take albendazole regularly. Only 22.9% took albendazole regularly. Of the 536 patients who took albendazole, 242 exhibited adverse reactions. Patients who were Tibetan, herdsmen, received no formal education, used emulsion, and exhibited adverse reactions demonstrated poor compliance. A total of 174 patients with cystic echinococcosis received their most recent imaging follow-up results within one year of the investigation date. Among them, 9 patients met the criteria for cure, accounting for 5.2%; 56 patients showed effectiveness, accounting for 32.2%; 105 patients were deemed ineffective, accounting for 59.8%; 5 patients experienced recurrence, accounting for 2.9%.

**Conclusions:**

Albendazole medication compliance in patients with echinococcosis is not ideal. We must prioritize health education and promotion for Tibetans, herdsmen, and those without formal education. Patients who adhered to their medication regimen achieved higher rates of cure and effectiveness. To improve medication compliance and efficacy, it is particularly important to improve communication and medication guidance for patients receiving emulsions and those with adverse reactions after taking albendazole. Simultaneously strengthen patients' attention to follow-up and re-examination.

**Graphical Abstract:**

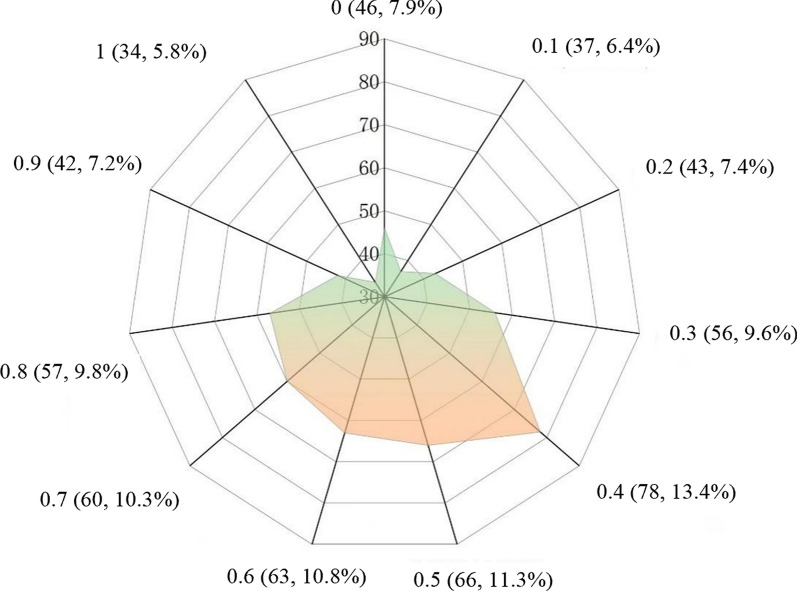

## Background

Echinococcosis is a parasitic disease that poses a major threat to human health, making it a global public health concern [1]. According to 2011 estimates, approximately 200,000 new patients are diagnosed with echinococcosis worldwide every year, with a total of 200 to 300 million patients [2]. The global burden of echinococcosis is 871,000 disability-adjusted life years (DALYs) annually [3]. Two types of echinococcosis are found in China: cystic echinococcosis (CE) and alveolar echinococcosis (AE). China is the country with the most severe endemic of the disease [4, 5]. The DALYs of CE and AE in China account for 40% and 95% of the world totals, respectively [6, 7]. Echinococcosis is a chronic, space-occupying disease [8, 9]. The lesions can accumulate in numerous organs, with the liver being the most prevalent site [10]. The CE cyst grows slowly, with an average incubation period of 5 to 10 years [11, 12]. As the cyst grows, it compresses the surrounding tissues, organs, and blood vessels, causing accompanying symptoms [10]. AE causes more severe damage. The lesions are invasive, infecting surrounding tissues and organs before metastasizing through lymphatic or blood vessels, hence its name of “worm cancer” [12]. If the disease is untreated or partially treated, the fatality rate will exceed 90.0% within 10–15 years [13, 14].

The treatment methods include surgery and medication. Medication is the most effective alternative treatment for patients who cannot undergo surgery [15]. The most commonly used therapeutic medications are benzimidazole drugs, specifically albendazole and mebendazole, which are effective at decreasing parasite growth and stabilizing disease [16, 17]. The World Health Organization recommends albendazole as the first line of clinical treatment [17]. Patients with echinococcosis who follow medical instructions and take medication regularly have a significantly increased life expectancy [18]. In albendazole treatment, patient adherence to taking medication regularly is key to preventing lesion recurrence and disease control [19].

In 2006, China launched the national project for echinococcosis control and prevention, which provides free albendazole tablets and emulsions to patients in need of medical treatment to reduce the economic burden. Patients undergo different treatment durations owing to the location, type, and size of the cyst, as well as other criteria (reexamination and liver function results), and the dosage is determined by the health service personnel based on body weight [20]. Tablets and emulsions have dosages of 15 and 0.8 ml/(kg·d), respectively, and are taken twice daily [20]. Although albendazole is a clinically effective therapeutic medication, its low bioavailability necessitates prolonged use [21]. Albendazole tolerance varies among patients. Some patients are intolerant of albendazole and will have adverse reactions if taken for an extended period, affecting medication compliance [21].

Therefore, monitoring the state of patient medications and their therapeutic impact will help improve medication compliance. To provide a valuable reference for designing appropriate intervention strategies and community health services, we examined the files of 899 patients with echinococcosis who were treated with albendazole in 10 endemic counties of China.

## Methods

### Data source

Random cluster sampling was used to select 10 echinococcosis-endemic counties (Daofu, Ganzi, Ruoergai in Sichuan Province; Huining, Tianzhu, Zhang, Maqu in Gansu Province; and Gaochang District, Jimusar, and Fukang City in Xinjiang Uygur Autonomous Region). We examined all patient files treated with albendazole that were registered with the Centers for Disease Control and Prevention in these counties. These patients all need to take the prescribed treatment drug albendazole, and divided into tablets and emulsions. Tablets and emulsions have dosages of 15 mg/(kg·d) and 0.8 ml/(kg·d), respectively, and are taken twice daily [22].

### Investigation contents

#### Patient medication

In 2019, data were collected by reviewing case files and conducting telephone follow-ups to collect general demographic data (sex, age, nation, education level, and occupation), disease type, diagnosis date, medication initiation date, dosage forms, occurrence of adverse reactions, and reasons for not taking medication.

Using medical records and follow-up information, we determined the theoretical duration of albendazole therapy. We evaluated albendazole compliance by comparing the actual and theoretical durations of albendazole therapy (R_m_).

Based on the distribution of R_m_, we defined regular medication use as R_m_ ≥ 0.8 and irregular medication use as R_m_ < 0.8. Albendazole therapy duration was measured in months and excluded if it was less than 15 days.

#### Adverse reactions of albendazole

According to the Diagnosis and treatment protocol for echinococcosis (2017 edition) [22], severity was determined based on the adverse reactions. Mild symptoms include a mild headache, dizziness, stomach discomfort, anorexia, nausea, diarrhea, skin itching, and liver acupuncture-like pain. Moderate symptoms include an aggravation of the aforementioned symptoms, vomiting, and a significant decrease in food intake. Severe symptoms include the aforementioned symptoms, noticeable alopecia, anemia, edema, jaundice, and a significant increase in bilirubin levels, decreased albumin and white blood cells, and increased albuminuria and creatinine levels revealed by laboratory examinations. Patients with mild symptoms usually do not need treatment and may continue taking albendazole. Moderate responders should discontinue albendazole use; it is recommended that they seek confirmation from a hospital and establish a treatment plan after blood, urine routine, and liver and kidney function tests. Severe responders should discontinue albendazole immediately and be referred to hospitals for treatment, if necessary.

#### Therapeutic efficacy

Patient records were reviewed to collect the initial diagnosis results and the most recent follow-up examination results and dates, and therapeutic efficacy assessments were conducted. Patients who had received imaging follow-up within one year of the investigation date were selected, with a focus solely on patients with CE owing to the limited number of patients with AE. Based on the most recent B-ultrasound examination results, the following criteria were used to determine the therapeutic efficacy for patients with CE (with lesions located in abdominal organs and the abdominal cavity) in accordance with the Technical Scheme for Drug Treatment of Hydatid Disease [22]:

I. Cure: disappearance of the cyst; complete calcification of the cyst wall; solidification of cyst contents.
II. Effective: a cyst diameter reduction of over 2.0 cm; signs of inner membrane detachment; increased echogenicity within the cyst contents, characterized by more pronounced bright spots.
III. Ineffective: no changes or progressive enlargement of the lesion.
IV. Recurrence: discovery of new lesions.

### Statistical analysis

The counting data are presented using frequency and constituent ratios. The comparison between groups was performed using the chi-square, Kruskal–Wallis, chi-square trend, and Fisher’s exact tests. *P* < 0.05 was considered statistically significant. The influencing factors were analyzed using multivariable logistic regression. The dependent variable was whether to take albendazole regularly. The estimated correlation strength was confirmed by the odds ratio (OR) with a 95.0% confidence interval (*CI*). Study participants were standardized by sex, age, education, occupation, and other patient-specific characteristics (Table 1). EpiData (version 3.1, EpiData Association, Odense, Denmark) was used to collect relevant information and establish a database. Data analysis was performed using SPSS software (version 22.0, IBM Corporation, Armonk, US).

**Table 1:** Variable standards

Variables	Attributes
Sex	Male = 1, Female = 2
Age	< 18 years old = 1, [18–40) = 2, [40–60) = 3, ≥ 60 years old = 4
Nation	Han nationality = 1, Tibetan = 2, Others = 3
Education	No formal education = 1, Primary = 2, Junior = 3, Senior = 4, College and above = 5
Occupation	Herdsman = 1, Farmer = 2, Others = 3
Disease type	CE = 1, AE = 2
Disease duration (year)	< 5 = 1, [5–10) = 2, ≥ 10 = 3
Albendazole dosage form	Tablet = 1, Emulsion = 2, Tablet + emulsion = 3
Occurrence of adverse reactions	Yes = 1, No = 2

*CE* cystic echinococcosis; *AE* alveolar echinococcosis

### Quality control

During the on-site investigation, specially assigned individuals in each county were responsible for reviewing the questionnaire and establishing quality control archives to ensure the authenticity and reliability of the data. During the data processing phase, missing values, outliers, and logic errors were rechecked, cleaned, and reprocessed.

## Results

### Patient medication

Three hundred and seventeen files lacked initial diagnostic and follow-up information; hence, we were unable to compute their actual and theoretical albendazole therapy durations, making medication evaluation impossible. Therefore, we evaluated 582 cases, accounting for 64.7%: 563 patients with CE (96.7%) and 19 patients with AE (3.3%). There were 368 females (63.2%) and 214 males (36.8%), with a sex ratio of 1.7:1. The maximum age was 92 years, and the minimum was 11 years. The average age was 64 years. In terms of age, 58.2% (339/582) of the patients were 60 years or older, while 30.6% (178/582) were 40–59 years old. The Tibetan population was the largest, followed by the Han population, accounting for 50.3% (293/582) and 46.1% (268/582), respectively. Farmers accounted for 49.1% (286/582) of the population, followed by herders at 42.1% (245/582). The majority had no formal education, accounting for 48.8% (284/582). The longest disease duration lasted 55 years, and the shortest lasted three months. The duration was concentrated in 5–9 years, accounting for 72.5% (422/582), as illustrated in Table 2.

**Table 2:** Albendazole medication in patients with echinococcosis in the 10 endemic counties in 2019

General characteristics	Total (*n*)	Medication			*H*	*P*-value
		No medication (*n*, %)	Irregular medication (*n*, %)	Regular medication (*n*, %)		
Sex						
Male	214	10 (4.7)	155 (72.4)	49 (22.9)	4.12	0.12
Female	368	36 (9.8)	248 (67.4)	84 (22.8)		
Age (year)						
< 18	13	3 (23.1)	8 (61.5)	2 (15.4)	3.89	0.14
18–40	52	1 (1.9)	36 (69.2)	15 (28.9)		
40–60	178	15 (8.4)	127 (71.4)	36 (20.3)		
≥ 60	339	27 (8.0)	232 (68.4)	80 (23.6)		
Ethnicity						
Han	268	1 (0.4)	193 (72.0)	74 (27.6)	34.76*	< 0.05
Tibetan	293	44 (15.0)	191 (65.2)	58 (19.8)		
Others	21	1 (4.2)	19 (90.5)	1 (4.2)		
Education level						
No formal education	353	38 (10.8)	224 (63.5)	91 (25.8)	46.12**	< 0.05
Primary school	164	6 (3.7)	133 (81.1)	25 (15.2)		
Junior high school	62	2 (3.2)	45 (28.6)	15 (24.2)		
Senior high school and above	3	0	1 (33.3)	2 (66.7)		
Occupation						
Herdsmen	245	37 (15.1)	156 (63.7)	52 (21.2)	31.69	< 0.05
Farmers	286	5 (1.8)	206 (72.0)	75 (26.2)		
Others	51	4 (7.8)	41 (80.4)	6 (11.7)		
Disease type						
CE	563	46 (8.2)	403 (71.6)	114 (20.3)	10.76	< 0.05
AE	19	0	0	19 (100.0)		
Disease duration (year)						
< 5	91	22 (24.2)	25 (27.5)	44 (48.4)	6.13	< 0.05
5–10	422	23 (5.5)	329 (78.0)	70 (16.6)		
≥ 10	69	1 (1.5)	49 (71.0)	19 (27.5)		
Albendazole dosage form						
Tablet	200	0	150 (75.0)	50 (25.0)	8.70	< 0.05
Emulsion	178	3 (1.7)	146 (82.0)	29 (16.3)		
Tablet + emulsion	111	0	63 (56.8)	48 (43.2)		
Unrecorded	93	43 (46.2)	44 (47.3)	6 (6.5)		
Occurrence of adverse reactions (Excluding patients without medication, N = 536)						
Yes	242	–	207 (85.5)	35 (14.5)	6.24	< 0.05
No	294	–	194 (66.0)	100 (34.0)		
Total	582	46 (7.9)	403 (69.2)	133 (22.9)		

The chi-square test compared only with the recorded percentage

*CE* cystic echinococcosis; *AE* alveolar echinococcosis

^*^Fewer patients from other ethnic groups and only Tibetan and Han patients were included in the analysis

^**^Fewer patients in senior high school, college, and beyond, and these patients were merged in the analysis

^†^*P* values were compared with the 0.05 level

**Fig. 1:**
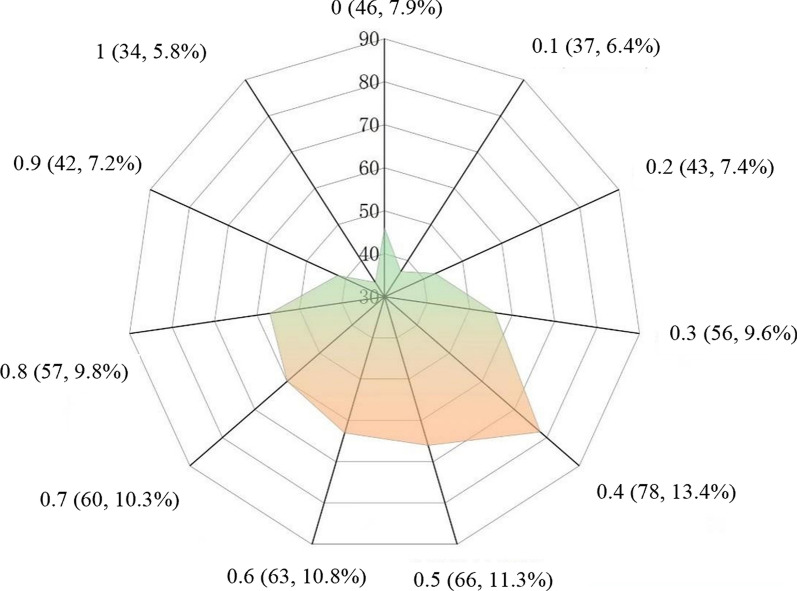
Distribution of albendazole compliance in patients with echinococcosis

As displayed in Fig. 1, R_m_ = 0.4 was the most concentrated, accounting for 13.4% (78/582), followed by R_m_ = 0.5, accounting for 11.3% (66/582). Only 22.9% of patients exhibited R_m_ ≥ 0.8. Stratification based on the theoretical duration of albendazole treatment showed R_m_ ≥ 0.8 in 90.5% (19/21), 87.5% (42/48), 75.6% (34/45), 13.5% (26/193), 4.6% (12/261), and 0.0% (0/14) patients taking albendazole for < 1 year, 1 year, 3 years, 5 years, 7 years, and ≥ 10 years, respectively. The proportion of patients with R_m_ ≥ 0.8 decreased with increased albendazole treatment duration (*χ*^*2*^_*trend*_ = 339.94, *P* < 0.05) (Table 3).

**Table 3:** Medication for Chinese patients with echinococcosis treated with albendazole in the 10 endemic counties in 2019, stratified by the duration of albendazole therapy

Duration of albendazole therapy (year)	Total (*N*)	Medication (*N*, %)				
		R_m_ = 0	0 < R_m_ < 0.3	0.3 ≤ R_m_ < 0.5	0.5 ≤ R_m_ < 0.8	R_m_ ≥ 0.8
< 1	21	1 (4.7)	0 (0.0)	0 (0.0)	1 (4.7)	19 (90.5)
1	48	3 (6.3)	0 (0.0)	0 (0.0)	3 (6.3)	42 (87.5)
3	45	1 (2.2)	0 (0.0)	1 (2.2)	9 (20.0)	34 (75.6)
5	193	6 (3.1)	20 (10.3)	56 (29.0)	85 (44.0)	26 (13.5)
7	261	31 (11.9)	54 (20.7)	73 (28.0)	91 (34.9)	12 (4.6)
≥ 10	14	4 (28.6)	6 (42.9)	4 (28.6)	0 (0.0)	0 (0.0)
Total	582	46 (7.9)	80 (13.8)	134 (23.0)	189 (32.5)	133 (22.9)
*χ*^*2*^		339.94				
*P*-value		*P* < 0.05				

Among the patients, 7.9% (46/582) had never taken albendazole; 69.2% (403/582) took it irregularly, and 22.9% (133/582) took it regularly. The percentage of regular medication varied significantly by ethnicity, education level, occupation, disease type, duration, and medication dosage form (*P* < 0.05). The details are presented in Table 2.

### Analysis of the factors influencing adherence to albendazole use

Multivariate logistic regression analysis revealed that Tibetan patients exhibited an OR of 0.06 (95% *CI* 0.03–0.12) for regular medication compared with Han patients. Herdsmen exhibited an OR of 0.08 (95% *CI* 0.04–0.15) for regular medication compared with farmers. Senior high school patients exhibited an OR of 1. 4 (95% *CI* 1.09–2.90) for regular medication compared with those without any formal education. Patients taking emulsions exhibited an OR of 0.46 (95% *CI* 0.22–0.93) for regular compared with those taking tablets. Patients without adverse reactions exhibited an OR of 1.81 (95% *CI* 1.02–3.20) for regular medication compared with those with adverse reactions. The results are detailed in Table 4.

**Table 4:** Multifactorial logistic regression analysis of the factors influencing albendazole-taking compliance in the 10 endemic counties in 2019

Variables	B	SE	Wald *χ*^*2*^	*P*	OR	95% *CI*
Ethnicity (ref. Han)						
Tibetan	− 2.79	0.32	74.94	< 0.05	0.06	0.03–0.12
Occupation (ref. Farmer)						
Herdsmen	− 2.56	0.33	61.49	< 0.05	0.08	0.04–0.15
Education level (ref. no formal education)						
Senior high school	0.57	0.25	5.22	< 0.05	1.77	1.09–2.90
	− 0.78	0.36	4.26	< 0.05	0.46	0.22–0.93
Occurrence of adverse reactions (ref. yes) no	0.59	0.29	4.17	< 0.05	1.81	1.02–3.20

Of the 403 patients who took albendazole irregularly, the main reasons for interrupted medication are as follows: 45.9% for adverse reactions, 26.3% for improving symptoms, 23.3% for doubting the efficacy of albendazole, 4.7% for intolerance, 4.5% for lack of access to drugs for school, work, or travel, and 3.5% owing to missed appointments.

Of the 46 patients who did not take the medication, 58.7% refused because of distrust in the efficacy of albendazole, and 41.3% refused because of intolerance to albendazole.

Of the 536 patients who took albendazole, 242 exhibited adverse reactions, with an incidence of 45.2%. The majority of adverse reactions were mild, accounting for 85.1% (206/242). The rates for moderate and severe were 12.0% (29/242) and 2.9% (7/242), respectively. Patients without adverse reactions displayed a significantly higher regular medication rate than those with adverse reactions (*H* = 26.93; *P* = 0.04) (Table 2).

### Analysis of the impact of patient medication compliance on therapeutic outcomes

The most recent imaging follow-up results from 174 patients with CE within one year of the investigation data were obtained. Nine patients met the cure criteria, accounting for 5.2%; 56 patients were considered to have been effectively treated, accounting for 32.2%; 105 patients were assessed as having shown no improvement, accounting for 59.8%; and 5 patients experienced recurrence, accounting for 2.9%. The actual treatment duration ranged from less than 1 month to 65 months, with an average of 18.7 ± 1.9 months. Patients experienced better treatment outcomes with regular medication use (*χ*^*2*^_*trend*_ = 0.280;* P* < 0.001). Of the 65 patients who received regular treatment, 13.9% were cured, 53.9% were effectively treated, and 32.3% showed no improvement. Of the 93 patients who did not receive regular treatment, 22.6% were effectively treated, 73.1% showed no improvement, and 4.3% experienced recurrence. Of the 16 patients who did not receive albendazole treatment, 93.8% showed no improvement, 6.3% experienced recurrence, and no patient was cured or effectively treated. Patients with higher medication adherence demonstrated better treatment outcomes (*χ*^*2*^_*trend*_ = 0.47;* P* < 0.05). The details are indicated in Table 5.

**Table 5:** Treatment outcomes of medication on patients with cystic echinococcosis

General condition	Total	Treatment outcome(*n*, %)				*χ*^*2*^_*trend*_	*P*
		Cured	Effective	Ineffective	Relapsed		
Actual albendazole administration duration (months)							
< 6	34	0	7 (20.6)	27 (79.4)	0	0.28	< 0.05
6–12	30	0	7 (23.3)	22 (73.3)	1 (3.3)		
12–36	43	3 (7.0)	15 (34.9)	23 (53.5)	2 (4.7)		
≥ 36	67	6 (9.0)	27 (40.3)	32 (47.8)	2 (3.0)		
Medication							
No medication	16	0	0	15 (93.8)	1 (6.3)	0.47	< 0.05
Irregular medication	93	0	21 (22.6)	68 (73.1)	4 (4.3)		
Regular medication	65	9 (13.9)	35 (53.9)	21 (32.3)	0		

In the stratified analysis based on patient treatment duration, among 34 patients who received less than 6 months of medication, 20.6% were effectively treated, while 79.4% showed no improvements, with no patients achieving a cure or experiencing recurrence. Among these patients, 13 took albendazole regularly, of whom 30.8% were effectively treated, while 15.0% of the 20 patients who took it irregularly were effectively treated. No statistically significant differences in treatment outcomes were observed among patients with different medication statuses (*Fisher* = 1.69*; P* = 0.52). Data are presented in Table 6.

**Table 6:** Analysis of medication and treatment outcomes of patients who took medication for less than six months

Treatment Effect	Total	No medication (n, %)	Irregular medication (n, %)	Regular medication (n, %)	*Fisher/χ*^*2*^_*trend*_	*P*
< 6						
Effective	7 (20.6)	0	3 (15.0)	4 (30.8)	1.69*	0.52
Ineffective	27 (79.4)	1 (100.0)	17 (85.0)	9 (69.2)		
6–12						
Effective	7 (23.3)	/	2 (11.1)	5 (41.7)	2.16*	0.21
Ineffective	22 (73.3)	/	15 (83.3)	7 (58.3)		
Relapsed	1 (3.3)	/	1 (5.6)	0		
12–36						
Cured	3 (7.0)	/	0	3 (20.0)	0.59	< 0.05
Effective	15 (34.9)	/	6 (21.4)	9 (60.0)		
Ineffective	23 (53.5)	/	20 (71.4)	3 (20.0)		
Relapsed	2 (4.7)	/	2 (7.1)	0		
≥ 36						
Cured	6 (9.0)	0	2 (7.4)	4 (16.0)	0.64	< 0.05
Effective	27 (40.3)	0	8 (29.6)	19 (76.0)		
Ineffective	32 (47.8)	14 (93.3)	16 (59.3)	2 (8.0)		
Relapsed	2 (3.0)	1 (6.7)	1 (3.7)	0		

^*^Fisher

For the 30 patients who received medication for 6–11 months, 23.3% were effectively treated, 73.3% showed no improvement, and 3.3% experienced relapse, with no patients achieving a cure. Among these patients, 12 took albendazole regularly, of whom 41.7% were effectively treated, while 11.1% of the 18 patients who took it irregularly were effectively treated. However, no statistically significant differences in treatment outcomes were observed among patients with different medication statuses (*Fisher* = 2.16;* P* = 0.21). Data are displayed in Table 6.

For the 43 patients who received medication for 12–35 months, 7.0% were cured, 34.9% were effectively treated, and 53.5% showed no improvement, with 4.7% experiencing recurrence. Among these patients, 15 took albendazole regularly, of whom 20.0% were cured, 60.0% were effectively treated, and 20.0% were ineffectively treated. However, of the 28 patients who took albendazole irregularly, 21.4% were effectively treated, 71.4% were ineffectively treated, and 7.1% experienced recurrence. Patients with higher medication adherence demonstrated better treatment outcomes (*χ*^*2*^_*trend*_ = 0.59;* P* < 0.05) (Table 6).

For the 67 patients who received medication for 36 months or more, 9.0% were cured, 40.3% were effectively treated, and 47.8% showed no improvement, with 3.0% experiencing recurrence. Among these patients, 25 took albendazole regularly, of whom 16.0% were cured, 76.0% were effectively treated and 8.0% were ineffectively treated. However, of the 27 patients who took albendazole irregularly, 7.4% were cured, 29.6% were effectively treated, 59.3% were ineffectively treated and 3.7% experienced recurrence. Additionally, the B-ultrasound results of 15 patients who did not receive medication showed an ineffective rate of 93.3% and a recurrence rate of 6.7%. Patients with higher medication adherence displayed better treatment outcomes *(χ*^*2*^_*trend*_ = 0.64; *P* < 0.05). The details are displayed in Table 6.

## Discussion

### Medication compliance

Drug therapy is the primary treatment option, especially for patients who cannot be treated surgically. Cyst becomes inactive or disappears after 1–2 years of albendazole treatment for CE, and patient compliance affects the long-term treatment outcome [20]. If liver lesions were removed and long-term benzimidazole treatment was used, the survival rates of patients with AE would be comparable to those of healthy people [23]. For patients with AE who cannot be treated with radical surgery, drug chemotherapy has significantly improved the 10-year survival rate from a range of 6.0%–25.0% to a range of 80.0%–85.0% [24].

The files of the patients were incomplete, preventing 35.3% of them from being evaluated for medication. These patients might have been lost to follow-up, or their medication information might not have been recorded owing to negligence by follow-up personnel [25]. The medication compliance of the evaluable patients was poor, and 7.9% had never taken albendazole. The majority of patients exhibited satisfactory adherence to medication during the early stages of treatment. However, the medication compliance of patients decreased as a result of the prolonged use of albendazole. Patients with echinococcosis demonstrated poor compliance with albendazole owing to poor knowledge of the disease, albendazole treatment, and adverse reactions [26, 27].

In this study, Tibetans and herdsmen without formal education comprised the majority of patients who failed to comply with medication. Patients with higher education levels, health awareness, and disease cognition are more likely to actively accept drug treatment. Recently, patients with a short course of disease have been diagnosed with echinococcosis, and their symptoms are more noticeable. Their compliance with medication is higher than that of patients with a long course of the disease. Patients with a long course of the disease feel relaxed because they believe their symptoms have improved. Patients with AE demonstrated higher medication compliance than patients with CE because their symptoms were more noticeable, and the disease was more severe. Additionally, the number of patients with AE in this study was small. AE is more complex and severe than CE, requiring long-term or even lifelong medication. This may lead to poor medication adherence among some patients, who may give up taking medication midway. It is important to follow up with these patients and remind them to take medication and undergo follow-up examinations regualarly.

Albendazole emulsion has an unpleasant taste and is unpalatable to some patients. However, it has a higher absorption rate than tablets and can improve treatment effectiveness [28]. We discovered that patients who combined tablets and emulsions exhibited higher compliance, implying that the patients preferred to choose their own suitable dosage form. The occurrence of adverse reactions was the main factor that affected patient medication compliance. The findings revealed that 85.1% of patients experienced mild adverse reactions and needed no treatments. However, 45.9% of patients discontinued taking albendazole owing to adverse reactions, indicating that patients exhibited poor knowledge of adverse reactions. We compared the results with those of other studies. Patients with echinococcosis exhibited poor compliance with albendazole owing to poor knowledge of the disease, albendazole treatment, and adverse reactions, indicating that the patients did not know how to deal with the adverse reactions properly. This suggests that patient health education on adverse reactions should be increased.

Additionally, patients who did not trust or pay attention to albendazole treatment, nor did understand treatment duration, exhibited poor medication compliance. Some Tibetan patients trusted traditional Tibetan medicine more and preferred to take local Tibetan medicine. Additionally, patients with echinococcosis are mainly herdsmen, especially Tibetans, who engage in nomadism [29]. During summer, they may be unable to obtain albendazole when they are nomadic in remote pastures. Consequently, herders have greater difficulty obtaining medications than farmers. Furthermore, Tibetan areas are located on the Qinghai-Tibet Plateau, making transit difficult. Some patients are unable to take albendazole on time because they are away or attending school.

We also suggest that disease control should work with clinical doctors to develop different treatment plans and follow-up times for patients based on their conditions, and judge the patient's medication time based on these indicators. For example, the Tibetan community, especially herdsmen, should be able to appropriately extend their follow-up interval and distribute more doses of medication. At the same time, a treatment group can be established within the nomadic community to supervise each other's medication, and a healthy supervisor can also be set up. After being informed of the characteristics of the two dosage forms, patients select the matching dosage form based on their sensitivity and convenience to avoid waste. Health education needs to focus on introducing the treatment of echinococcosis, especially the characteristics and necessity of drug therapy, as well as the effectiveness of albendazole treatment. It should also be emphasized that the characteristics of the two dosage forms to patients, as well as the possible adverse reactions, symptom manifestations, and treatment methods of albendazole, in order to alleviate patients’ fear of albendazole. Follow up and follow-up examinations are important means of reminding patients to take medication and evaluating the effectiveness of their treatment, which requires their cooperation. In health education, it is also necessary to emphasize the necessity of follow-up and re examination in drug treatment, which is related to their course of treatment.Treatment groups can also be established based on the patient's village or community, and healthcare professionals can be arranged to answer their questions, remind patients to take medication or undergo follow-up examinations. Health education should pay special attention to patients with relatively low levels of education, who may not be familiar with or even have misunderstandings about the treatment of echinococcosis and albendazole drugs, and may not value the importance of follow-up and re examination. These are the key to providing health education to them, and patients who take medication regularly and have good treatment effects can be invited as promoters to educate them.

Medication compliance may vary depending on factors such as disease duration, severity, and type. In this study, patients in ten echinococcosis-endemic counties from three endemic provinces in China were investigated for medication compliance, with good representativeness; however, it did not include all epidemic counties.

### Therapeutic efficacy

Cure and effectiveness rates were 5.2% and 32.2%, respectively, which are lower than previously reported data where the cure rate of albendazole treatment for patients was approximately 30.0%, with an effectiveness rate ranging from 40.0% to 60.0% and an inefficacy or recurrence rate of 30.0% [30]. This may be attributed to the fact that only patients with follow-up records within the past year were included in this study, while follow-up information for other patients who were medication-compliant was not documented, making it impossible to evaluate their treatment outcomes. In this study, patients who adhered to regular medication displayed considerably better treatment outcomes than those who did not adhere to medication or did not take medication, consistent with findings from other studies [30, 31]. This implies the need for improved patient health education to enhance medication adherence, as albendazole therapy is a long-term procedure, especially for certain cystic types that remain active. This will increase the disease and economic burden on patients. For patients who cannot undergo surgical treatment, standardized medication is the most cost-effective and only treatment option. Regular medication can improve patient treatment outcomes, indicating that relevant departments should increase the intensity and quality of patient follow-up and health education, ensure effective follow-up work implementation, and improve patient medication adherence to further improve cure rates and quality of life.

In this study, the effectiveness and cure rates for patients who had taken medication for more than 36 months were 40.3% and 9.0%, respectively, which are lower than those reported by Li et al., where the effectiveness rate of albendazole treatment for patients with CE after 3–6 months was 30.0%. When treatment was extended for 18–30 months, the number of cysts decreased by 32.7%, and cystic degeneration decreased by 49.0%, yielding an overall effectiveness rate of 81.0% [30]. Due to its low absorption rate in the intestine and low concentration of active ingredients in the capsule, albendazole requires an increased dosage to achieve therapeutic effects. However, high-dose medication can cause some adverse reactions, leading to poor medication compliance and liver damage, causing additional physical damage and economic burden to patients. The treatment of CE is related to the size, classification, and quantity of the patient's lesions. However, in this study, no stratified analysis was conducted on the characteristics of patient lesions.

A small number of patients obtained follow-up results within the previous year, making it difficult to assess patient treatment outcomes. Therefore, only 174 patients were evaluated for treatment effectiveness, reflecting the problems in the management of patients with CE medication treatment as well as poor compliance and a lack of emphasis on follow-up.

### Suggestions

We recommend the following: First, relevant departments must improve the quality of patient management and record-keeping. Second, it is necessary to focus on patient health education to increase their knowledge of the disease, treatment, and reexamination, especially the performance and treatment of albendazole-related adverse reactions, to reduce the fear of adverse reactions. Drug therapy is a long-term procedure that requires patients to regularly re-examine and evaluate its efficacy. Third, follow-up personnel should receive more professional and comprehensive training, and they should be required to fully document each follow-up. Albendazole must be dispensed in a flexible manner. After being informed of the characteristics of the two dosage forms, patients select the matching dosage form based on their sensitivity and convenience to avoid waste. Regarding patient nomadism, follow-up personnel should keep open communication with patients, regularly assess the existing albendazole stock of patients, leave a buffer time for drug distribution, and avoid the situation of insufficient drugs for patients.

More extensive and diversified health education and professional guidance should be provided to patients receiving medical treatment in community health services to improve their knowledge of the harm caused by echinococcosis, albendazole treatment of echinococcosis, adverse reactions, and follow-up management, thereby increasing their trust and confidence in albendazole treatment and alleviating their fear of adverse reactions. Their families and community doctors and officials should be invited to attend. Actively implementing regular management for patients with echinococcosis undergoing medication therapy can increase their adherence to regular treatment, as well as delay and prevent progression and recurrence. Tertiary prevention can increase cure rates, decrease disability and mortality rates, and improve their quality of life.In this study, the medical files of some patients might not have documented timely information about their albendazole use. Additionally, some patients might have had recall bias during the investigation, resulting in an underestimation of medication compliance.

### Limitations

The limitations of this study include a sample size and a large number of excluded cases. First, 35.3% of the research patients corresponding to the files were excluded from the analysis, which could have resulted in selection bias that could not be evaluated. Second, surveying 10 out of the 370 endemic counties in China resulted in low representativeness. Third, in the analysis of the treatment effect of CE patients, the inclusion of too few patients may lead to selection bias and cannot represent the actual treatment situation of patients. These biases can lead to discrepancies between research results and actual situations. In future research, we recommend conducting long-term follow-up on patients to obtain more accurate information on their medication use and treatment outcomes. Additionally, it is recommended to include more patients in future analyses. There may be differences in health education strategies in different regions. Therefore, we aim to conduct a uniform nationwide special survey in the future.

## Conclusions

In conclusion, patients demonstrated poor compliance with albendazole use, highlighting the need for improved health education and medication guidance, as well as more follow-up to improve compliance. This study recommends that medication awareness should be emphasized in community health services to improve knowledge of echinococcosis drug therapy and albendazole's adverse effects. Additionally, it is necessary to strengthen patients' attention to follow-up and re-examination. Regular medication can improve patient treatment effectiveness, suggesting that relevant departments should intensify their efforts and enhance the quality of patient health education. This ensures that follow-up and re-examination are conducted effectively, patient medication adherence is improved, and patient's cure rates and quality of life are increased.

## Data Availability

All data analyzed in the present study are included in the article materials. Any inquiries can be directed to the corresponding author.

## Abbreviations

AE: Alveolar echinococcosis
CE: Cystic echinococcosis
TAR: Tibet Autonomous Region
DALYs: Disability-adjusted life years
CDC: Center for disease control and prevention
R_m_: The actual duration of albendazole therapy and the theoretical duration of albendazole therapy
